# Care-seeking and appropriate treatment for childhood acute respiratory illness: an analysis of Demographic and Health Survey and Multiple Indicators Cluster Survey datasets for high-mortality countries

**DOI:** 10.1186/1471-2458-14-446

**Published:** 2014-05-12

**Authors:** Emily M Mosites, Alastair I Matheson, Eli Kern, Lisa E Manhart, Saul S Morris, Stephen E Hawes

**Affiliations:** 1 Department of Global Health START Program, University of Washington, 325 9th Avenue, Seattle, WA, USA; 2 The Bill & Melinda Gates Foundation, 500 5th Ave N, Seattle, WA, USA

**Keywords:** Acute lower respiratory infection, Antibiotic treatment, Child health, High mortality countries

## Abstract

**Background:**

Acute lower respiratory illness (ALRI) is a major global cause of morbidity and mortality among children under 5. Antibiotic treatment for ALRI is inexpensive and decreases case fatality, but care-seeking patterns and appropriate treatment vary widely across countries. This study sought to examine patterns of appropriate treatment and estimate the burden of cases of untreated ALRI in high mortality countries.

**Methods:**

This study used cross-sectional survey data from the Phase 5/Phase 6 DHS and MIC3/MICS4 for 39 countries. We analyzed care-seeking patterns and antibiotic treatment based on country-level trends, and estimated the burden of untreated cases using country-level predictors in a general linear model.

**Results:**

According to this analysis, over 66 million children were not treated with antibiotics for ALRI in 2010. Overall, African countries had a lower proportion of mothers who sought care for a recent episode of ALRI (41% to 86%) relative to Asian countries (75% to 87%). Seeking any care for ALRI was inversely related to seeking public sector care. Treatment with antibiotics ranged from 8% in Nepal to 87% in Jordan, and was significantly associated with urban residence.

**Conclusions:**

Untreated ALRI remains a substantial problem in high mortality countries. In Asia, the large population numbers lead to a high burden of children with untreated ALRI. In Africa, care-seeking behaviors and access to care issues may lead to missed opportunities to treat children with antibiotics.

## Background

According to the 2010 Global Burden of Disease estimates, acute lower respiratory infection (ALRI) is the leading cause of death among children aged under five years in developing countries [1]. Although viruses are the more common etiologic agent of ALRI in children [2], bacterial pathogens are associated with higher risk of morbidity and mortality [3] and viral ALRIs are often followed by bacterial infections [4]. While two of the most common etiologies of bacterial ALRI, *Haemophilus influenza* type b (Hib) and *Streptococcus pneumoniae,* can be partially prevented through vaccination, vaccine coverage is not yet widespread in resource-limited settings [5]. Vaccines are not available for any of the other bacterial pathogens that cause ALRI. Until effective vaccines become broadly available and vaccination coverage is high, proper clinical management remains the most important method of preventing childhood mortality from ALRI.

Appropriate clinical management of ALRI consists of several elements: a caregiver recognizing the signs and symptoms of severe infection, the child being brought to a healthcare facility, the healthcare worker making a correct diagnosis, and the availability of appropriate therapy. Each of these components provides one or more pathways by which a child with ALRI might not receive appropriate treatment.

As a clinical syndrome, ALRI is characterized by fever, cough, difficult breathing, and rapid breathing although each of these may be variable in young children and difficult for caregivers to evaluate. In one analysis, only 20% of caregivers were able to identify difficulty breathing and fast breathing as characteristic symptoms [6].

Severity of ALRI upon presentation predicts response to treatment [7], and therefore prompt care-seeking by the parents of ill children is critical. However, delays in care-seeking often occur and can be related to cost, non-recognition of the severity of illness, differences in the perception of the cause of disease, or geographic barriers [8-11]. Additionally, poor healthcare infrastructure and/or lack of appropriate care and medicine contribute to delays [12].

Correct diagnosis of ALRI is often difficult in areas that are endemic for diseases with similar clinical presentations, such as tuberculosis or malaria [13]. Additionally, severity must be accurately assessed and antibiotic treatment and oxygen therapy provided, if necessary. The World Health Organization (WHO) currently recommends intramuscular ampicillin and gentamycin for inpatients with very severe pneumonia, and oral amoxicillin or co-trimoxazole for less severe pneumonia and outpatients [14]. Other common antibiotics for severe ALRI include oral cephalosporins and macrolides [15]. When needed, antibiotic therapy has dramatic effects on child survival; case-fatality rates for treated ALRI are under 1% while untreated ALRI case-fatality rates may reach over 10% [16]. However, there are a number of barriers to receiving appropriate antibiotics at the clinic level, including stock-outs, understaffing, prescribing patterns of health workers, and diagnostic overlaps with other infections [11,12,17,18].

Given these challenges, both parental care-seeking and treatment patterns within facilities vary widely across countries. The WHO has estimated that globally, only 30% of children with ALRI are appropriately treated with antibiotics [19], though this proportion is likely to be even lower in high-mortality countries.

To effectively reduce the burden of ALRI mortality, it is important to understand patterns of care-seeking, as well as to quantify the number of cases of untreated ALRI within high mortality countries. However, direct comparisons among countries with high mortality are made difficult by a lack of routinely collected estimates that use similar methodology. Two exceptions to this are the Demographic and Health Surveys (DHS) and Multiple Indicator Cluster Surveys (MICS). These household-level surveys measure health and demographic indicators approximately every 5 years in developing countries using nationally representative samples. They are particularly useful for aggregate estimation of burden because they use a common, standardized approach. DHS data provides information on fertility and health topics, while the MICS is focused on the state of women and children to inform policy and program decisions. DHS and MICS surveys have been intentionally developed to be comparable to each other.

To date, no study has aggregated DHS and MICS indicators to compare ALRI care-seeking and treatment outcomes globally. Though DHS and MICS data are the best source of comparable data, many high-mortality countries have not conducted a DHS or MICS. Fortunately it is possible to use the information from countries which have been surveyed to provide information on those which have not yet been surveyed. This study uses the most recent DHS and MICS data to address two objectives: 1) gain an improved understanding of patterns of care-seeking and antibiotic treatment among countries with DHS and MICS data, and 2) determine whether country-level factors can be used to estimate the burden of cases of untreated ALRI in all high-mortality countries.

## Methods

### Datasets

DHS datasets (phases 5 and 6) from African and Asian countries were accessed from http://www.measuredhs.com/Data/. Datasets were included in the analysis if they contained data points for the components of a restricted ALRI case definition, and the components of either care-seeking behavior or antibiotic treatment (see below). MICS datasets (MICS3 and MICS4) for Asian and African countries were accessed from http://www.childinfo.org/mics3_surveys.html. MICS antibiotic treatment data were accessed through the country final reports [20]. MICS country report information was included if it contained the sufficient data on antibiotic treatment (total and stratified by urban/rural). Estimates of the number of respiratory infection cases in each country were calculated using under-5 population and pneumonia prevalence data from the Child Health Epidemiology Research Group (CHERG), which were recently updated to account for vaccine roll-out [21]. Country-level predictors of the proportion treated with antibiotics were collated from WHO and World Bank datasets [5,22].

Data management and analysis were conducted using Stata SE 11 (Stata Corp, LP). All data were publically available and not identifiable; therefore ethical review was not required.

### Definitions

DHS and MICS datasets are designed to be highly comparable, and questions on the topic of ALRI were asked in the same manner in both surveys. In this analysis, for both datasets, *ALRI* was defined as “child under age five who was ill in the previous two weeks with a cough accompanied by short, rapid breathing which the mother considered to be chest-related”. *Care-seeking* was defined as “mother sought advice or treatment for this illness from any source”. The variables for *seeking medical care* or *seeking public sector care* were defined as a composite of answers to the question “the place at which medical treatment or advice was sought for the last episode of fever and/or cough”, the answers to which were not mutually exclusive categories. *Seeking medical care* specifically involved seeking care at an appropriate medical provider, either in the private or public sector, not including pharmacies. *Antibiotic treatment* was defined as an affirmative answer to the question “at any time during the illness, the child took an antibiotic pills/syrup or injection for this illness”, or an antibiotic was listed in the “other” or country-specific field of the question “what drugs did *name* take?” Urban or rural residence in the DHS was defined by each country’s census data [23].

### Country-level descriptive analyses

In order to describe the ALRI treatment patterns across the countries with available data, we calculated the proportions of care-seeking and antibiotic treatment within each country. These estimates were weighted using the standard sampling weights which are provided in the DHS and MICS data. The sampling weights account for each survey design, which included over- and under-sampling of particular regions and allow the calculation of overall national estimates.

We calculated four quantities for each country: (a) the proportion of children with ALRI who received any care (# for whom care was sought/total # of ALRI cases); (b) proportion of children with ALRI who received any *medical* care (# children for whom medical care was sought/total # of ALRI cases); (c) proportion of children with ALRI who received medical care from a public facility (# of children for whom public care was sought/# for whom care was sought); and (d) proportion of children with ALRI who received antibiotic treatment (# of children who received antibiotics/total # of ALRI cases).

The proportion of children receiving any care was plotted against the proportion of children receiving public sector care. This trend was evaluated for significance using robust standard errors to account for heteroscedasticity.

### Prediction model for number of untreated ALRI cases

We developed a prediction model to estimate the proportion of children who were not treated for ALRI in 80 high-mortality countries for which DHS or MICS data were not available. To create this model, we generated a dataset of 28 countries that did have available data on the country-level proportion of antibiotic treatment in children with ALRI from the DHS or MICS (year of data collection ranging from 2006 to 2011). We then evaluated the following independent variables for their association with antibiotic treatment proportion: proportion of women receiving at least one antenatal care visit; coverage of the third dose of the diphtheria, pertussis, and tetanus vaccine in one-year olds; coverage of Bacillus Calmette-Guérin (BCG) vaccine in one-year-olds; coverage of measles-containing virus (MCV) vaccine in one-year-olds; gross national income (GNI) per capita; health expenditures per capita (PPP); number of nurses/midwives per 1,000 people; percentage of the population living in urban areas; coverage of oral rehydration salts for the treatment of diarrhea; infant mortality per 1,000 live births per year; under-five mortality per 1,000 live births per year; age-adjusted total mortality per 1,000 people per year; and an indicator of African or Asian continent. To test these associations, we conducted univariate analyses using pairwise linear correlation coefficients. Independent variables with a correlation coefficient above 0.3 were included as potential predictors in a multivariate general linear model.

The final model was chosen through a backwards step-wise selection process based on Akaike information criterion (AIC) values. The model included analytic weights based on the standard errors of the DHS and MICS survey estimates of the dependent variable, to account for point estimate variability in the original surveys. The selected model was then subjected to leave-one-out cross-validation to estimate overall mean squared error. This model was used to predict proportions of ALRI that went untreated for all countries with predictor data. Predictions were limited to countries with child and overall mortality rates that lay within the bounds of the countries with DHS or MICS data, to avoid extrapolation. Finally, the predicted proportions were applied to CHERG estimates of the number of ALRI cases in each country to obtain an overall estimate of the absolute number of children who are not treated for respiratory illness each year.

## Results

As of August 2012, 17 countries within Africa and Asia had DHS datasets with information about care-seeking patterns for ALRI among children under 5 years of age. Eighteen datasets containing care-seeking information were available from the MICS datasets. For the predictive model, eleven countries had DHS datasets with information about antibiotic treatment of ALRI (two of which did not have care-seeking data), while 15 additional countries had antibiotic treatment information in MICS reports.

### Care-seeking patterns

**Figure 1:**
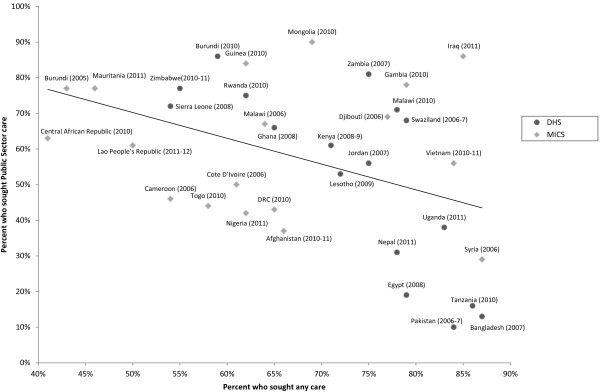
Proportions of children who received any care compared to those who received public sector care, Phase 5/6 DHS and MICS 3/4.

Access to care for children with ALRI was highly variable across countries with recent DHS or MICS datasets available, and ranged from 41% to 87% (Table 1). Overall, African countries had a lower percentage of mothers who sought care for a recent episode of ALRI (41% to 86%, median 64%) relative to Asian countries (75% to 87%, median 81%). Seeking medical care, specifically, was more common in African than Asian countries. Those countries with the highest percentage seeking non-medical care (i.e., seeking care from pharmacies, shops, and traditional healers) were Bangladesh, Nepal, and Swaziland. Overall, 10% to 86% sought care in the public sector for a child’s ALRI, and this was more common in African than Asian countries. In many of the African countries with data available, over 50% of care was sought in public healthcare facilities. There was a significant negative correlation between seeking public sector care and seeking any care at all (robust p = 0 · 01); in those countries with a low frequency of care-seeking overall, a higher percentage of individuals sought care in the public sector (Figure 1). Those countries with higher care-seeking had wider variability in accessing public sector care.

**Table 1:** Proportions of ALRI cases seeking care, per Phase 5/6 DHS and MICS 3/4

**Dataset**	**Country (year)**	Any care*	Medical care*	Public sector care**
DHS	Sierra Leone (2008)	54%	47%	72%
	Zimbabwe (2010–11)	55%	48%	77%
	Burundi (2010)	59%	56%	86%
	Rwanda (2010)	62%	50%	75%
	Ghana (2008)	65%	51%	66%
	Kenya (2008–9)	71%	56%	61%
	Lesotho (2009)	72%	66%	53%
	Jordan (2007)	75%	75%	56%
	Zambia (2007)	75%	69%	81%
	Nepal (2011)	78%	50%	31%
	Malawi (2010)	78%	72%	71%
	Swaziland (2006–7)	79%	58%	68%
	Egypt (2008)	79%	73%	19%
	Uganda (2011)	83%	73%	38%
	Pakistan (2006–7)	84%	71%	10%
	Tanzania (2010)	86%	72%	16%
	Bangladesh (2007)	87%	57%	13%
MICS	Central African Republic (2010)	41%	32%	63%
	Mauritania (2011)	46%	39%	77%
	Lao People’s Republic (2011–12)	50%	40%	61%
	Cameroon (2006)	54%	35%	46%
	Togo (2010)	58%	32%	44%
	Cote D’Ivoire (2006)	61%	35%	50%
	Guinea (2010)	62%	56%	84%
	Nigeria (2011)	62%	35%	42%
	DRC (2010)	65%	39%	43%
	Afghanistan (2010–11)	66%	60%	37%
	Mongolia (2010)	69%	62%	90%
	Djibouti (2006)	77%	58%	69%
	Gambia (2010)	79%	77%	78%
	Vietnam (2010–11)	84%	73%	56%
	Iraq (2011)	85%	82%	86%
	Syria (2006)	87%	73%	29%

*Among all ALRI cases.

**Among those who sought any care.

### Antibiotic treatment patterns

In the 26 countries with DHS or MICS estimates available, treatment with antibiotics for children with a recent episode of ALRI ranged from 8% in Nepal to 87% in Jordan (Table 2). Urban residents had an average of 10 percentage points higher levels of antibiotic treatment compared to rural residents (paired p-value for difference < 0 · 001).

**Table 2:** Percent of ALRI cases receiving antibiotics, according to Phase 5/6 DHS and MICS 3/4

**DHS**				**MICS**			
**Country (year)**	**Overall**	**Urban**	**Rural**	**Country (year)**	**Overall**	**Urban**	**Rural**
Nepal (2011)	8%	9%	8%	Yemen (2006)	13%	12%	13%
Swaziland (2006–7)	16%	18%	15%	Cote D’Ivoire (2006)	19%	26%	16%
Ghana (2008)	24%	29%	21%	Mozambique (2008)	22%	29%	19%
Namibia (2006–7)	36%	52%	31%	Mauritania (2007)	24%	30%	19%
Zambia (2007)	47%	66%	39%	Togo (2006)	26%	27%	25%
Kenya (2008–9)	50%	46%	50%	Malawi (2006)	30%	38%	28%
Pakistan (2006–7)	51%	56%	49%	Central African Republic (2010)	31%	47%	23%
Uganda (2011)	51%	59%	50%	Somalia (2006)	32%	49%	24%
Liberia (2011)	54%	62%	51%	Cameroon (2006)	38%	58%	27%
Egypt (2008)	58%	63%	54%	Guinea (2006)	42%	55%	33%
Jordan (2007)	87%	87%	91%	Djibouti (2006)	43%	43%	20%
				Gambia (2005)	61%	60%	62%
				Afghanistan (2010–11)	64%	70%	63%
				Mongolia (2005)	71%	72%	71%
				Syria (2006)	71%	75%	66%

### Prediction model

The prediction model for untreated ALRI is included in Table 3. In the selected model, the percent of the country that was urban and the under-five mortality rate were the only country-level characteristics that were retained. The model predicted the proportion of children with ALRI who were untreated for 108 countries. Across these 108 countries, the estimated number of children with ALRI who do not receive antibiotics exceeds 66 million per year, which represents 53% of ALRI cases (see Additional file 1 for list of country-specific data).

**Table 3:** Country-level general linear regression model predicting proportion of ALRI cases who were treated with antibiotics

**Covariate**	**Coefficient**	**P-value**	**95% CI**
Percent Urban	−0.00388	0.067	−0.0080 – 0.0003
Under 5 mortality*	0.105	0.030	0.0102 – 0.1998

*Log transformed (natural log) under-five mortality per 1,000 live births per year.

## Discussion

Antibiotic treatment is an inexpensive, effective way to increase child survival for children with ALRI in resource-limited settings. In this study, we estimated that over 66 million children in high-mortality countries who could benefit from antibiotic therapy are not treated, and that there is wide variability across countries in care-seeking and antibiotic treatment patterns for children with presumed ALRI.

Cultural and political factors may influence care-seeking behaviors. Some of the lowest proportions of parents seeking any care for their child’s illness were in Central African Republic, Burundi, and Sierra Leone. These were also the countries with some of the highest percentages of parents seeking public sector care. It is possible that the low level of care-seeking in these countries is related to a relative lack of private sector healthcare. Each of these countries has experienced extensive political instability which may have influenced the overall availability of care and the ability of parents to seek care. On the opposite side of the spectrum, Bangladesh, Tanzania, Syria and Pakistan each had high levels of access to care, but low percentages accessing public sector care. Although the private sector is well established, it is generally less regulated, and sales of inappropriate drugs are a recurrent problem [24].

In the countries with available DHS and MICS data, antibiotic treatment levels among children with presumed ALRI were quite low. There was wide variation in antibiotic coverage across countries, with the lowest percentage occurring in rural areas.

To our knowledge, no other studies have attempted to predict the burden of untreated pneumonia in high-mortality countries for which no publically available data exist. This prediction model provides meaningful estimates of the number of untreated children for mathematical models seeking to estimate how much disease could be resolved if treatment guidelines were followed, and how many prognoses can be improved. Although the DHS and MICS provide the most extensive methodologically comparable collection of datasets for global estimation, there are several limitations that may bias estimates of care-seeking and proportion of cases treated with antibiotics. The DHS data available spanned several years, so the model findings are most applicable to the years between 2005 and 2010. Due to the nature of self-report in the DHS, it is also likely that the DHS definition of ALRI is neither sensitive nor specific for radiographically confirmed pneumonia, and thus we would hope to observe fewer than 100% of cases treated with antibiotics [25]. Recent studies have posited that the antibiotic treatment estimates derived from DHS and MICS data cannot be accurate because of this low sensitivity and specificity of pneumonia recognition [26]. Furthermore, the DHS and MICS rely on maternal recall of antibiotic treatment, the challenges of which are well documented in the literature [26]. However, in this prediction model, we applied estimates of the proportion of children receiving antibiotics derived from DHS/MICS to CHERG estimates of pneumonia incidence, which is a more specific characterization of pneumonia burden than in the DHS. With large enough sample sizes, we expect that bias in the DHS/MICS antibiotic treatment proportions would only be introduced from differential treatment provision based on the severity of illness of those ALRI cases included in the DHS. The extent and direction of this bias is unclear, and further study is necessary.

The prediction model estimated that 66 million cases of ALRI go untreated every year. While the best model was fit from the data which were publically available, the predicted estimates from the linear model for countries with available DHS or MICS data were often much closer to the mean than the observed data. Because of this, the model is limited in its capacity to estimate the burden of untreated ALRI for individual countries. However, the model reflects improved estimates of the total burden for these high mortality countries by encompassing predictive indicators for countries without data. With the sampling variability of DHS estimations, particularly when combined with CHERG predictions, the estimates for each country have a wide predictive range; therefore the absolute number of untreated cases for each country should be interpreted with caution.

## Conclusions

ALRI is a major contributor to child mortality in developing countries. Our analysis shows that there are large numbers of children with ALRI who are not treated with antibiotics, representing an enormous missed opportunity to prevent mortality. While the WHO estimated that globally 30% of children are untreated for pneumonia, our data suggest that, in high mortality countries, over 50% of children may be untreated. It is likely that this high proportion of untreated children is adding to the overall mortality rate of these countries. Additionally, there is likely unnecessary usage of antibiotics which, given the multibillion dollar global market in antibiotics, represents wasted funds that could be redirected to support evidence-based health care practices. Integrated initiatives such as community case management of pneumonia have recently been demonstrated to be effective for appropriately providing treatment to children with pneumonia [27-30]. These community-based interventions promote both care-seeking and appropriate antibiotic treatment, and ultimately reduce mortality due to untreated ALRI.

## Competing interests

SM was employed by the Bill & Melinda Gates Foundation at the time of writing. Otherwise, the authors do not report any conflicts of interest.

## Authors’ contributions

EM, AM, and EK collected data, conducted the analyses and contributed to writing the manuscript. LM, SM, and SH contributed to the scientific design of the study and reviewed the manuscript. All authors read and approved the final manuscript.

## Pre-publication history

The pre-publication history for this paper can be accessed here:

http://www.biomedcentral.com/1471-2458/14/446/prepub

## Supplementary Material

Additional file 1 **CHERG ALRI estimates and predictive model output.** Country-level data table for the CHERG estimates of ALRI and the output of the predictive model demonstrating the burden of untreated ALRI. Click here for file
