# Supplementary material for: Care-seeking and appropriate treatment for childhood acute respiratory illness: an analysis of Demographic and Health Survey and Multiple Indicators Cluster Survey datasets for high-mortality countries
Source: BMC Public Health. 2014 May 12;14:446. doi: 10.1186/1471-2458-14-446 (PMC4024183; doi:10.1186/1471-2458-14-446)
Supplement: Additional file 1 — CHERG ALRI estimates and predictive model output. Country-level data table for the CHERG estimates of ALRI and the output of the predictive model demonstrating the burden of untreated ALRI. [file 1471-2458-14-446-S1.docx]

#### ADDITIONAL FILE 1: CHERG estimates and Predictive Model Output

| Country | 2010 adjusted CHERG ALRI estimate | Prediction Model | | |
| --- | --- | --- | --- | --- |
|  |  | Proportion not receiving ABX | Standard error | Number not receiving ABX |
| India | 35361230 | 0·62 | 0·04 | 21955699 |
| Nigeria | 7339761 | 0·63 | 0·06 | 4648390 |
| Pakistan | 6728235 | 0·63 | 0·04 | 4223006 |
| Bangladesh | 4484527 | 0·60 | 0·05 | 2691152 |
| DRC | 3671614 | 0·71 | 0·06 | 2591226 |
| Ethiopia | 3367561 | 0·73 | 0·06 | 2444588 |
| Indonesia | 3918360 | 0·47 | 0·04 | 1841720 |
| Afghanistan | 2040302 | 0·73 | 0·06 | 1498686 |
| Tanzania, United Republic of | 2151379 | 0·66 | 0·04 | 1411522 |
| Uganda | 1745727 | 0·73 | 0·06 | 1281808 |
| Sudan | 2061300 | 0·62 | 0·04 | 1274390 |
| Kenya | 1645189 | 0·68 | 0·05 | 1124253 |
| Philippines | 2428448 | 0·40 | 0·06 | 971099 |
| Viet Nam | 1728193 | 0·52 | 0·07 | 903582 |
| Niger | 1127652 | 0·76 | 0·06 | 854039 |
| Burkina Faso | 1047365 | 0·77 | 0·06 | 803888 |
| Mozambique | 1155781 | 0·67 | 0·05 | 774271 |
| Myanmar | 1213300 | 0·61 | 0·04 | 745152 |
| Yemen | 1150463 | 0·64 | 0·04 | 734101 |
| Mali | 932894 | 0·72 | 0·06 | 670123 |
| Madagascar | 1051407 | 0·62 | 0·04 | 651050 |
| Côte d'Ivoire | 985611 | 0·62 | 0·06 | 608611 |
| Nepal | 832451 | 0·64 | 0·06 | 535389 |
| Angola | 856794 | 0·61 | 0·08 | 523397 |
| Chad | 678297 | 0·74 | 0·06 | 500986 |
| Cameroon | 790160 | 0·59 | 0·07 | 468690 |
| Malawi | 658512 | 0·70 | 0·05 | 463129 |
| Somalia | 650669 | 0·70 | 0·06 | 458068 |
| Ghana | 795448 | 0·56 | 0·04 | 442581 |
| Brazil | 1497706 | 0·28 | 0·10 | 416298 |
| Iraq | 893131 | 0·43 | 0·06 | 384933 |
| Zambia | 576056 | 0·66 | 0·04 | 378533 |
| Guinea | 546525 | 0·68 | 0·05 | 370313 |
| Senegal | 591373 | 0·59 | 0·04 | 348207 |
| South Africa | 705554 | 0·49 | 0·05 | 345878 |
| Mexico | 1110027 | 0·30 | 0·08 | 329999 |
| Egypt | 680363 | 0·46 | 0·06 | 312991 |
| Rwanda | 397910 | 0·70 | 0·05 | 279392 |
| Iran, Islamic Republic of | 729564 | 0·38 | 0·07 | 274892 |
| Burundi | 349477 | 0·78 | 0·07 | 272551 |
| Benin | 424074 | 0·64 | 0·05 | 270377 |
| Cambodia | 373583 | 0·63 | 0·05 | 235253 |
| Sri Lanka | 433688 | 0·54 | 0·10 | 234842 |
| Guatemala | 481781 | 0·48 | 0·04 | 229385 |
| Haiti | 345081 | 0·65 | 0·07 | 225068 |
| Sierra Leone | 315676 | 0·70 | 0·06 | 219887 |
| Zimbabwe | 349031 | 0·61 | 0·04 | 214644 |
| Algeria | 470713 | 0·42 | 0·06 | 198918 |
| Morocco | 385554 | 0·46 | 0·05 | 177876 |
| Togo | 280487 | 0·62 | 0·04 | 174497 |
| Korea, DPR | 393494 | 0·43 | 0·05 | 167267 |
| Colombia | 488486 | 0·32 | 0·08 | 156607 |
| Eritrea | 208035 | 0·65 | 0·05 | 135721 |
| Central African Republic | 195417 | 0·68 | 0·06 | 133512 |
| Laos | 212441 | 0·60 | 0·04 | 126819 |
| Liberia | 212990 | 0·55 | 0·07 | 117645 |
| Syrian Arab Republic | 280849 | 0·38 | 0·07 | 106745 |
| Peru | 313170 | 0·33 | 0·07 | 104043 |
| Saudi Arabia | 337985 | 0·29 | 0·09 | 97267 |
| Mauritania | 144982 | 0·64 | 0·04 | 92459 |
| Congo, Republic of the | 168619 | 0·54 | 0·06 | 90674 |
| Honduras | 184407 | 0·45 | 0·05 | 82944 |
| Venezuela | 308502 | 0·24 | 0·11 | 74432 |
| Bolivia | 137114 | 0·47 | 0·06 | 63780 |
| Nicaragua | 141434 | 0·43 | 0·05 | 60430 |
| Ecuador | 163860 | 0·36 | 0·07 | 59132 |
| Turkey | 172393 | 0·34 | 0·07 | 58299 |
| Paraguay | 139661 | 0·40 | 0·06 | 56379 |
| Guinea-Bissau | 75199 | 0·71 | 0·05 | 53541 |
| Uzbekistan | 82133 | 0·57 | 0·03 | 47112 |
| Dominican Republic | 121820 | 0·38 | 0·07 | 45911 |
| Gambia | 79805 | 0·56 | 0·06 | 44900 |
| Kazakhstan | 94676 | 0·44 | 0·05 | 42080 |
| Lesotho | 58335 | 0·67 | 0·04 | 38959 |
| China | 88722 | 0·44 | 0·06 | 38602 |
| Namibia | 63796 | 0·55 | 0·04 | 34837 |
| Tunisia | 99837 | 0·33 | 0·07 | 33302 |
| Jordan | 87843 | 0·32 | 0·08 | 28493 |
| Mongolia | 60292 | 0·45 | 0·05 | 26837 |
| El Salvador | 72388 | 0·36 | 0·07 | 25830 |
| Comoros | 38380 | 0·66 | 0·04 | 25382 |
| Libya | 80748 | 0·30 | 0·08 | 24006 |
| Botswana | 47818 | 0·48 | 0·05 | 22764 |
| Swaziland | 28802 | 0·66 | 0·05 | 19087 |
| Tajikistan | 25144 | 0·64 | 0·05 | 16002 |
| Gabon | 36186 | 0·42 | 0·10 | 15224 |
| Jamaica | 31065 | 0·43 | 0·05 | 13370 |
| Azerbaijan | 23855 | 0·50 | 0·04 | 11989 |
| Djibouti | 24926 | 0·43 | 0·11 | 10835 |
| Equatorial Guinea | 16341 | 0·65 | 0·05 | 10696 |
| Lebanon | 35518 | 0·29 | 0·10 | 10281 |
| Kyrgyzstan | 17168 | 0·54 | 0·04 | 9349 |
| Turkmenistan | 14823 | 0·53 | 0·04 | 7929 |
| Bhutan | 12773 | 0·59 | 0·03 | 7476 |
| Trinidad and Tobago | 9784 | 0·59 | 0·09 | 5811 |
| Mauritius | 13518 | 0·42 | 0·07 | 5727 |
| Cape Verde | 9874 | 0·45 | 0·05 | 4402 |
| Guyana | 7186 | 0·55 | 0·06 | 3957 |
| Georgia | 7488 | 0·42 | 0·06 | 3154 |
| Sao Tome and Principe | 5118 | 0·53 | 0·06 | 2691 |
| Armenia | 6661 | 0·37 | 0·06 | 2455 |
| Suriname, Republic of | 6578 | 0·37 | 0·07 | 2447 |
| Belize | 4795 | 0·40 | 0·06 | 1909 |
| Maldives | 4061 | 0·44 | 0·08 | 1768 |
| Saint Lucia | 1492 | 0·48 | 0·08 | 723 |
| Bahamas | 2514 | 0·27 | 0·09 | 673 |
| Barbados | 1377 | 0·46 | 0·06 | 636 |
| Saint Vincent and Grenadines | 967 | 0·44 | 0·06 | 425 |
| Total | 110239629 | 0·53 | 0·06 | 66475060 |
